# Combined anti-S1 and anti-S2 antibodies from hybrid immunity elicit potent cross-variant ADCC against SARS-CoV-2

**DOI:** 10.1172/jci.insight.170681

**Published:** 2023-08-08

**Authors:** Michael D. Grant, Kirsten Bentley, Ceri A. Fielding, Keeley M. Hatfield, Danielle P. Ings, Debbie Harnum, Eddie C.Y. Wang, Richard J. Stanton, Kayla A. Holder

**Affiliations:** 1 Immunology and Infectious Diseases Program, Division of BioMedical Sciences, Faculty of Medicine, Memorial University of Newfoundland, St. John’s, Newfoundland, Canada.; 2 Division of Infection and Immunity, School of Medicine, Cardiff University, Cardiff, United Kingdom.; 3 Eastern Health Regional Health Authority, St. John’s, Newfoundland, Canada.

**Keywords:** Immunology, Vaccines, Cellular immune response, Immunoglobulins, NK cells

## Abstract

Antibodies capable of neutralizing SARS-CoV-2 are well studied, but Fc receptor–dependent antibody activities that can also significantly impact the course of infection have not been studied in such depth. Since most SARS-CoV-2 vaccines induce only anti-spike antibodies, here we investigated spike-specific antibody-dependent cellular cytotoxicity (ADCC). Vaccination produced antibodies that weakly induced ADCC; however, antibodies from individuals who were infected prior to vaccination (hybrid immunity) elicited strong anti-spike ADCC. Quantitative and qualitative aspects of humoral immunity contributed to this capability, with infection skewing IgG antibody production toward S2, vaccination skewing toward S1, and hybrid immunity evoking strong responses against both domains. A combination of antibodies targeting both spike domains support strong antibody-dependent NK cell activation, with 3 regions of antibody reactivity outside the receptor-binding domain (RBD) corresponding with potent anti-spike ADCC. Consequently, ADCC induced by hybrid immunity with ancestral antigen was conserved against variants containing neutralization escape mutations in the RBD. Induction of antibodies recognizing a broad range of spike epitopes and eliciting strong and durable ADCC may partially explain why hybrid immunity provides superior protection against infection and disease compared with vaccination alone, and it demonstrates that spike-only subunit vaccines would benefit from strategies that induce combined anti-S1 and anti-S2 antibody responses.

## Graphical abstract

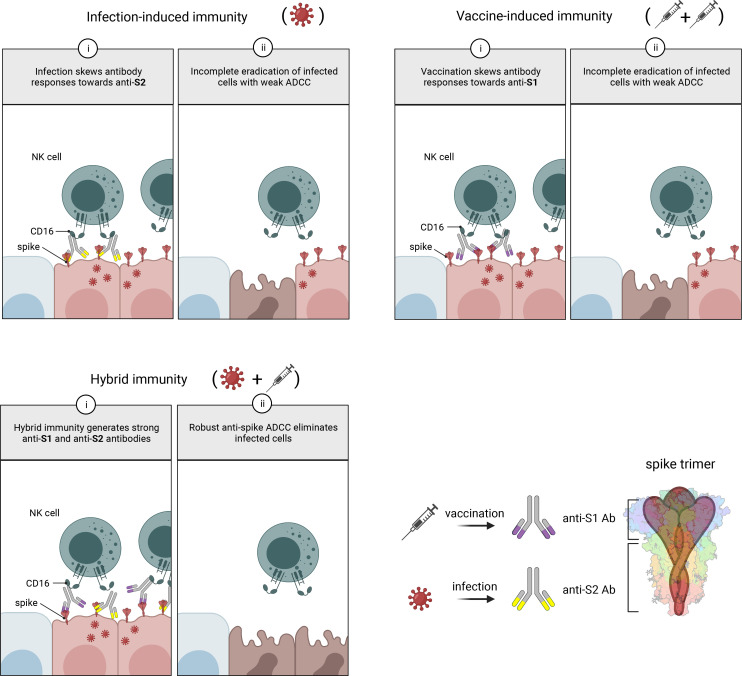

## Introduction

Humoral immunity against SARS-CoV-2 spike (S) protein is induced by infection and by vaccination with any of the predominant COVID-19 vaccines used worldwide, most of which encode S as a single antigen (1–3). Anti-S Abs target multiple regions within the protein, but the major focus has been on those that neutralize cell-free virions. These primarily bind within the receptor-binding domain (RBD) or, in some cases, the N-terminal domain (NTD), both of which are found in the S1 domain of the protein. Neutralizing Abs block or prevent binding between SARS-CoV-2 and the entry receptor angiotensin converting enzyme-2 (ACE-2) or prevent postbinding events required for virus entry (4, 5). They are thought to be crucial for reducing transmission of SARS-CoV-2; thus, they are a key measure for predicting COVID-19 vaccine efficacy (6).

Despite their clear importance, neutralizing Abs have recognized limitations. The number of neutralizing epitopes is limited, resulting in rapid selection of SARS-CoV-2 variants with mutations that weaken Ab binding to key neutralizing sites (7, 8). After approximately 3 years of evolution in the human population, SARS-CoV-2 variants of concern have largely escaped the neutralizing activity of Abs induced by the ancestral S antigen and are continually evolving to evade Abs induced by infection with more recent variants. As a result, the efficacy of vaccines at preventing infection is already reduced within months of their introduction. Once infection occurs, SARS-CoV-2 can undergo direct cell-to-cell transmission, further undermining the efficacy of neutralizing Ab (9).

To counteract cell-to-cell virus spread, Abs are required that, rather than neutralizing cell-free virions, recognize viral antigens on the surface of infected cells (10). These recruit effector cells such as NK cells to kill infected cells through Ab-dependent cellular cytotoxicity (ADCC), thereby controlling cell-associated virus. Infection with SARS-CoV-2 readily induces Abs capable of supporting ADCC (11), and ADCC is a key determinant of immunological control in animal challenge models (12–21). We and others have shown that Abs capable of ADCC are effective at preventing disease in animals even in the complete absence of neutralizing activity (18, 22). Thus, inducing and maximizing this activity through vaccination is highly desirable.

In addition to being a target for neutralizing Abs, SARS-CoV-2 S is also expressed on the infected cell surface, where it is efficiently bound by Abs (11). Thus, S has the potential to be an effective target for Fc receptor–mediated (FcR-mediated) NK cell activation, enabling a single-antigen vaccine capable of inducing Ab activity targeting both cell-free and cell-associated virus. Although vaccination induces anti-S Abs capable of activating NK cells when tested against purified protein (23–25) or transfected cells (26–31), our previous study revealed that, when tested against live virus, effective ADCC was dominated by Abs targeting nucleocapsid (N), membrane (M), and ORF3a, and individuals who had only anti-S Abs (i.e., vaccinees with no infection history) demonstrated weak ADCC (11).

Subsequent studies have made it clear that infection with SARS-CoV-2 prior to vaccination with a S-encoding vaccine (hybrid immunity) offers superior protection compared with vaccination or infection alone (32–35). To explore FcR-dependent mechanisms that may contribute to this protection, we examined quantitative and qualitative aspects of S-induced humoral immunity and its association with ADCC potency in individuals who had recovered from infection, been vaccinated, or both.

## Results

### Hybrid immunity elicits robust ADCC against S-transduced cells.

Since all donors in this study were vaccinated with SARS-CoV-2 S–encoding vaccines, we isolated S-specific ADCC from responses targeting other SARS-CoV-2 proteins by testing Abs for their capacity to mediate lysis of SARS-CoV-2 S–expressing target cells. A series of plasma dilutions were performed initially to establish optimal levels on a subset of samples, and plasma was diluted 1:1,000 for subsequent experiments. This dilution distinguished ADCC from no ADCC for vaccinee and postinfection samples and discriminated ADCC levels from hybrid immunity across a wide range of samples without plateauing.

**Figure 1:**
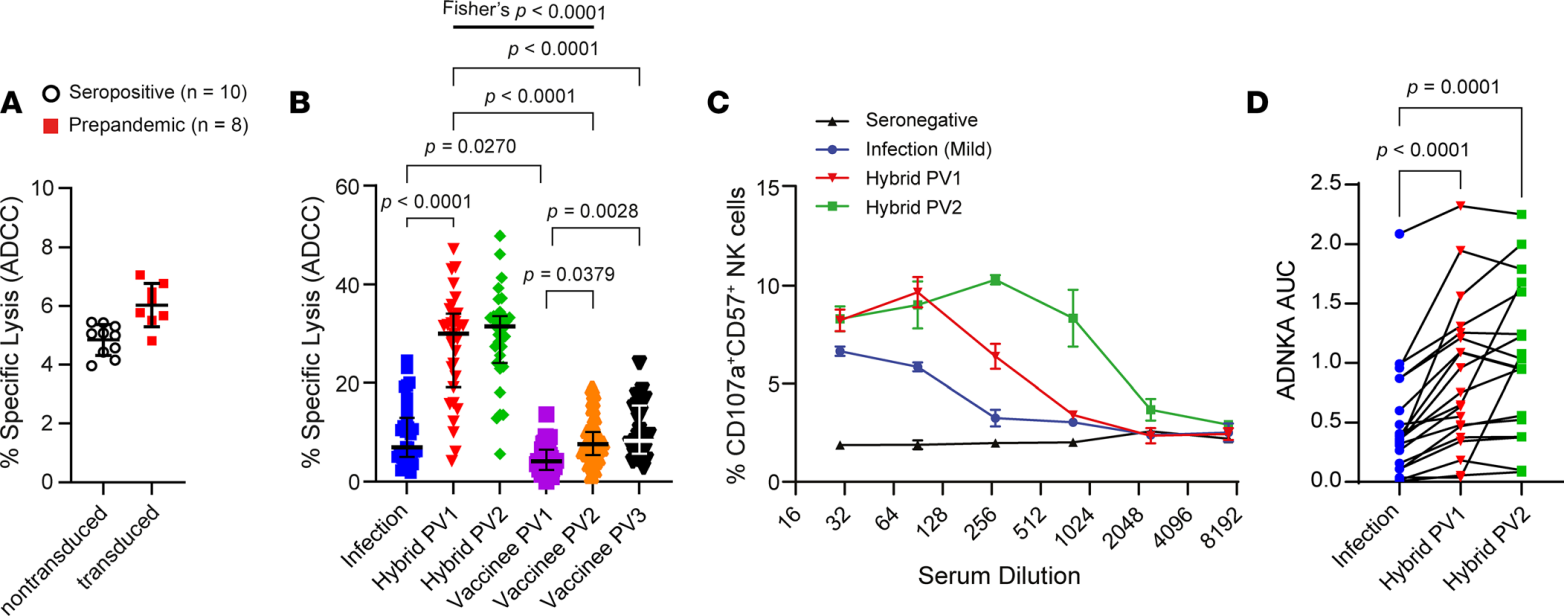
Vaccine-, infection-, and hybrid immunity–elicited S-specific NK cell activation. (**A**) Background NK cell lysis of nontransduced (open black circle) or Wu-S–expressing (red square) MRC-5 cells elicited by plasma from SARS-CoV-2 seropositive (*n* = 10) or plasma collected before pandemic (*n* = 8), respectively, was measured by ^51^Cr release (E:T, 25:1). (**B**) Sequential measures of Wu-S–MRC-5 cell ADCC elicited by plasma collected after infection then subsequent vaccination (hybrid *n* = 31) or after vaccination alone (vaccinee PV1 and PV2, *n* = 40; vaccinee PV3, *n* = 31) was measured by ^51^Cr release (E:T, 25:1). Experiments were performed in duplicate with 3 independent donors, and a representative plot shown. Vaccinee PV3 percent lysis data were collected after the initial data set and standardized. (**C** and **D**) Serum samples were serially diluted, and CD57^+^ NK cell CD107a degranulation against A549-ACE2 cells infected with SARS-CoV-2 twenty-four hours previously at MOI 5 was measured by flow cytometry. Data from a single individual are shown (**C**) and compiled data from multiple donors (*n* = 20) (**D**) was assessed by calculating the AUC. Lines bisecting groups represent the mean of individual plasma samples with SD (**A**), and represent median with IQR (**B**). *P* value in **B** was calculated using Kruskal-Wallis test with Dunn’s multiple-comparison test, and in **D**, it was calculated using 1-way ANOVA for matched data with Tukey’s correction. The probability of hybrid immunity after 1 vaccine inducing more robust ADCC than vaccine-induced immunity (2 vaccine doses) was calculated using 2-sided Fisher’s exact test.

There was minimal background ADCC against parental (nontransduced) MRC-5 elicited by SARS-CoV-2 seropositive plasma and against Wuhan-Hu-1 S–expressing MRC-5 (Wu-S–MRC-5) cells by prepandemic plasma (Figure 1A). Consistent with previous data (11), plasma from participants recovered from SARS-CoV-2 infection mediated weak S-specific ADCC (mean 7.0% lysis) with Abs from only 13 of 31 individuals inducing > 10% lysis (Figure 1B). Responses were weaker among those who were vaccinated and not previously infected, with only 1 participant mediating ADCC > 10% after 1 vaccination (PV1; Figure 1B). A second vaccination (PV2) increased responses slightly, with 10 of 40 individuals demonstrating killing above 10% specific lysis, while a third vaccination had no further impact on ADCC (Figure 1B). Thus, S-specific ADCC remained low overall, comparable with that of participants with infection-induced immunity (mean 7.7% lysis; Figure 1B). In contrast, hybrid immunity substantially enhanced ADCC (mean 31.3% lysis), with the absolute number of participants mediating > 10% lysis increasing to 29 of 31 after a single vaccination (Figure 1B). There was no further increase to ADCC within the hybrid group upon second vaccination; however, 1 additional participant (30 of 31) mediated > 10% ADCC (Figure 1B).

### Hybrid immunity induces significant S-specific ADCC against infected cells.

Although cells transfected or transduced to express S provide a platform to isolate S-specific ADCC, Ab-dependent NK cell responses in the context of virus infection can differ significantly from those with cells overexpressing surface protein (11). Since ^51^Cr release assays are incompatible with BSL3 conditions, we assessed Ab-dependent NK cell activation (ADNKA) against SARS-CoV-2–infected cells by measuring NK cell degranulation (surface CD107a expression) as a surrogate for ADCC.

During infection, ADNKA is dominated by Abs targeting antigens other than S, obscuring increases in S-specific ADNKA attributed to vaccination (11). To circumvent this issue, we focused on individuals with asymptomatic or mild infection (and, therefore, comparatively weak prevaccine ADNKA responses) followed by vaccination with a S subunit vaccine, and we tested sera over a range of dilutions. This allowed us to select a dilution at which infection-induced anti-N/M/ORF3a responses had faded but where a significant boost to S-specific ADNKA could be measured following vaccination (Figure 1C) across multiple donors (Figure 1D). Although a second vaccine dose enhanced the abundance of ADNKA-capable Abs in some donors, such that reactivity was maintained at higher dilutions (Figure 1C), it did not alter maximal levels of ADNKA (Figure 1, C and D). Therefore, anti-S Abs can mediate S-specific Ab-dependent NK cell activity against transduced or infected cells. However, these Abs are not efficiently induced by natural infection or vaccination; a hybrid combination is required.

### Neutralization and ADNKA engage different Ab populations.

**Figure 2:**
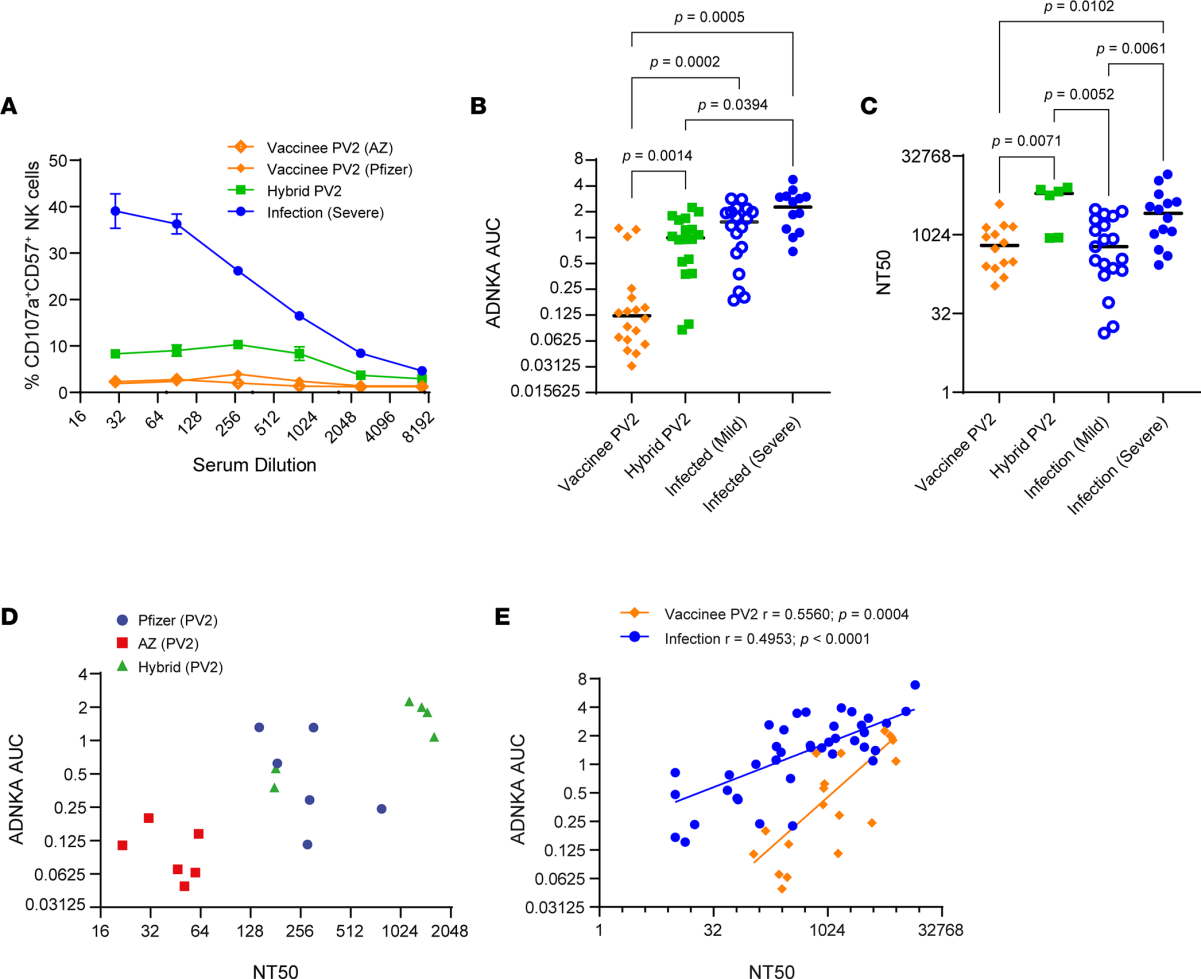
Spike-directed ADNKA against SARS-CoV-2–infected cells. (**A** and **B**) A549-ACE2 cells were infected with SARS-CoV-2 for 24 hours, and CD57^+^ NK cell CD107a expression was measured in response to serial dilutions of sera from vaccinees (PV2), hybrid immunity (PV2), or persons recovered from mild or severe infection and no vaccination. Representative data in **A** are depicted at the indicated dilutions, and in **B**, the AUC was calculated and data compiled for multiple donors (vaccinee, *n* = 18; hybrid immunity, *n* = 18; mild infection, *n* = 18; severe infection, *n* = 14). (**C**) Sera used in **B** were applied to SARS-CoV-2–infected cells, neutralization assessed, and NT50 calculated. *P* values in **B** and **C** were calculated using 1-way ANOVA with Tukey’s correction. Lines bisecting groups in **A**–**C** represent mean ± SD. (**D** and **E**) The significance of correlations between ADNKA and NT50 were assessed using Spearman’s correlation.

We next used infected cells to compare S-specific ADNKA mediated by hybrid immunity induced by mild or severe infection to determine whether S-specific ADNKA provided by hybrid immunity approached the level of ADNKA induced by the sum of Abs targeting all SARS-CoV-2 cell-surface proteins (i.e*.*, including anti-N/M/ORF3a; Figure 2A) (11). Individuals were further stratified by the vaccine they received (adenovirus [AstraZeneca ChAdOx1-S] or mRNA [Pfizer-BioNTech]). Vaccination alone induced weak ADNKA, irrespective of vaccine platform (Figure 2, A and B). In contrast, hybrid immunity boosted S-specific ADNKA to levels comparable with the potent multiantigen ADNKA of individuals who had recovered from mild infection but not to the extent of those recovered from severe infection (Figure 2B). This pattern was in sharp contrast to the neutralizing Ab response, in which vaccination induced responses comparable with mild infection and hybrid immunity gave responses comparable with severe infection (Figure 2C).

To investigate further, we considered the correlation between neutralizing and ADNKA Ab titers against live virus. Comparisons among vaccinated individuals in which ADNKA was primarily driven by anti-S Ab responses demonstrated a positive correlation between neutralization and ADNKA, along with a clear hierarchy of responses. Vaccination with adenovirus (AstraZeneca ChAdOx1-S) demonstrated the weakest neutralization and ADNKA activity, mRNA (Pfizer-BioNTech) vaccination induced better ADNKA and neutralization than adenovirus vaccination, and both vaccination regimes were inferior to neutralization and ADNKA induced by hybrid immunity (Figure 2D). Although neutralization and ADNKA showed positive correlations in vaccinees and infected individuals (11), the relationship between the 2 activities was markedly different. For any given level of neutralization, infection showed superior levels of ADNKA compared with vaccination (Figure 2E). Thus, in comparison with infection, S-based vaccines were effective at inducing neutralizing Abs and less so at inducing ADNKA, consistent with the presence of Abs against multiple different ADCC targets following infection.

### Superior ADCC from hybrid immunity is not strictly explained by Ab abundance.

To test whether differences in ADCC between hybrid and vaccine-induced immunity were due in part to IgG abundance, we compared ADCC with measurements of total IgG targeting full-length S (FLS) and individual S1 and S2 subunits. Comparisons were also carried out on IgG_3_ subclass Abs; although IgG_1_ is most abundant, IgG_3_ supports more potent ADCC (36, 37).

**Figure 3:**
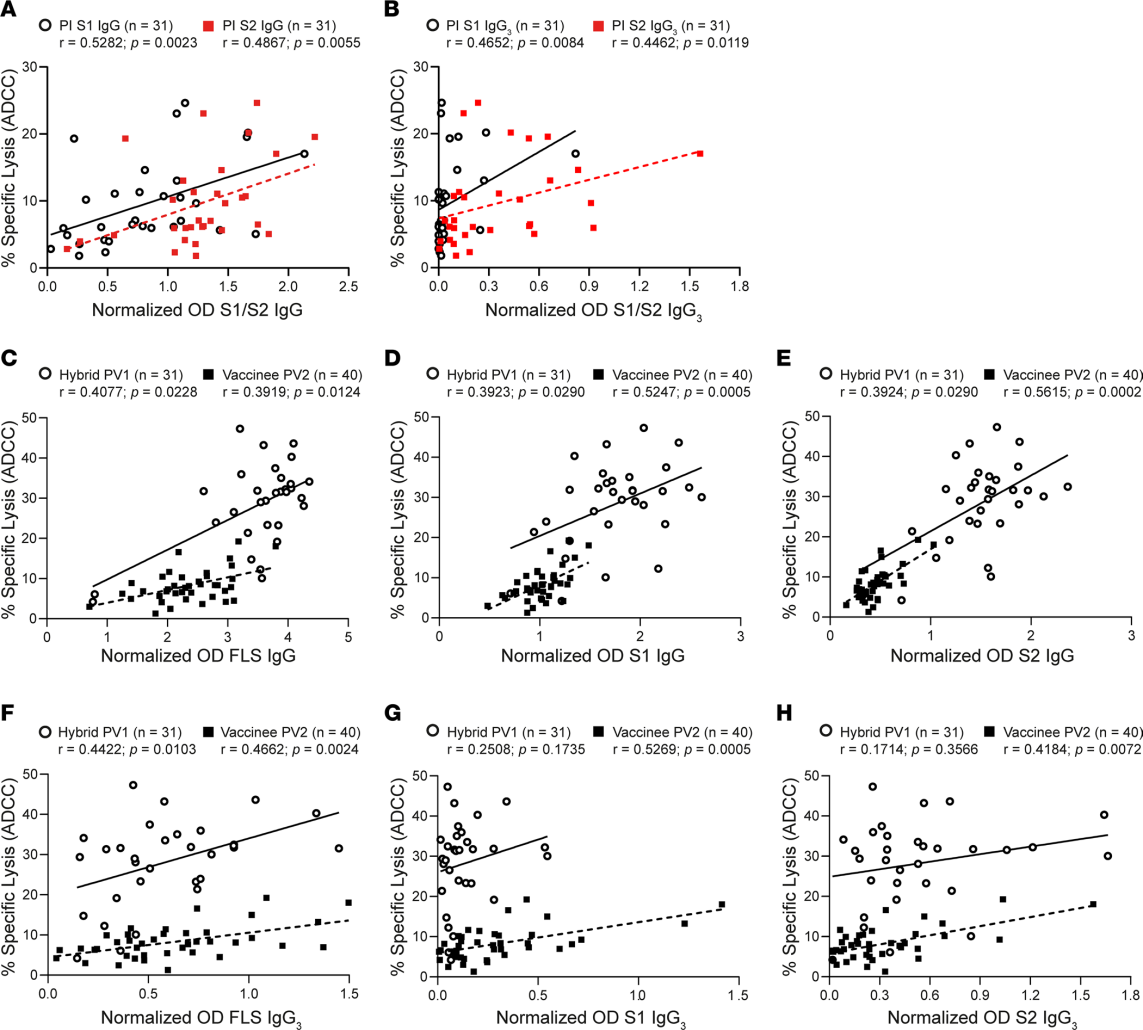
Associations between ADCC and anti–S1/S2 IgG and IgG_3_ Ab abundance. (**A** and **B**) Spearman’s correlations between ADCC induced by plasma Ab from samples collected postinfection (PI) and levels of anti–S1 IgG (open black circle) and anti–S2 IgG (red square) as well as IgG_3_ are plotted. (**C**–**H**) Relationships between the magnitude of ADCC mediated by hybrid (PV1; open black circle; *n* = 31) and vaccinee (PV2; black square; *n* = 40) plasma Ab and anti-FLS IgG, anti–S1 IgG, and anti–S2 IgG or anti-FLS IgG_3_, anti–S1 IgG_3_ and anti–S2 IgG_3_ are depicted.

ADCC positively correlated with amounts of anti–S1 and anti–S2 IgG (Figure 3A) and IgG_3_ (Figure 3B) Abs, despite anti–S1 IgG_3_ not being abundant in those recovered from infection (Figure 3B). Since the hybrid group demonstrated more robust ADCC after first vaccination and there were no significant increases in the levels of Abs or ADCC after second vaccination (see below), we focused on responses elicited from the hybrid group after 1 vaccine to contrast with the vaccinees after second vaccination. In both the hybrid cohort (PV1) and the vaccine group (PV2), ADCC correlated with increased anti–FLS IgG Ab levels (Figure 3C). This trend persisted when we compared anti–S1 IgG (Figure 3D) and anti–S2 IgG (Figure 3E) Ab levels with ADCC. There was also a significant correlation between levels of anti–FLS IgG_3_ Ab and ADCC in both the hybrid and vaccine cohorts (Figure 3F), although this correlation was lost for the hybrid group when comparing reactivity against individual S1 and S2 subunits of SARS-CoV-2 S (Figure 3, G and H). This may reflect that the lower abundance of IgG_3_ results in IgG_1_ having a greater influence on total ADCC activity.

Despite these strong correlations between Ab levels and ADCC within cohorts, there were significant differences between the cohorts, with Abs from vaccinees eliciting much weaker ADCC for a given level of Abs compared with their hybrid counterparts. Thus, although Ab levels influence ADCC activity, Ab abundance alone does not explain the superior ADCC induced by hybrid immunity.

### Vaccination, infection, and hybrid immunity induce distinct Ab responses to S1 and S2.

**Figure 4:**
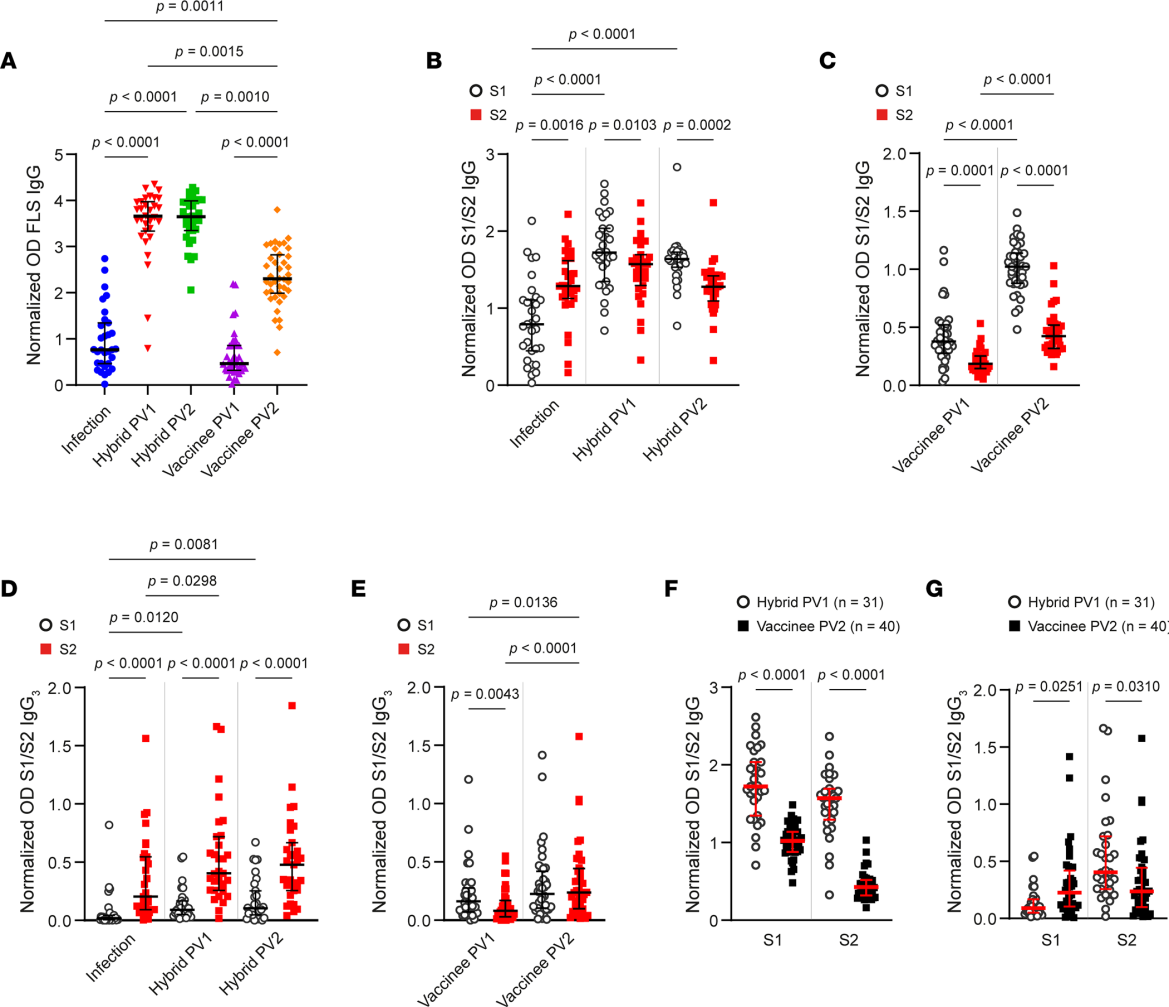
Anti-S1/S2 Ab responses after vaccination, infection, and hybrid immunity. (**A**) Circulating anti-FLS IgG Abs from participants with hybrid immunity were measured postinfection (PI; blue circle) and after first (PV1; red chevron) and second (PV2; green square) vaccinations as well as from vaccinated participants (*n* = 40) PV1 (purple triangle) and PV2 (orange diamond). (**B** and **C**) Levels of anti–S1 IgG (open black circle) and anti–S2 IgG (red square) were compared for participants with hybrid immunity (PI, PV1, and PV2) and vaccinees (PV1, and PV2). (**D** and **E**) Relative amounts of anti-S1 and anti–S2 IgG_3_ were measured for persons with hybrid immunity or vaccinees, and anti–S1 IgG_3_ and anti–S2 IgG_3_ Ab levels were compared. (**F** and **G**) Hybrid- and vaccine-induced anti–S1 and anti–S2 IgG and IgG_3_ were contrasted. *P* values in **A**, **F**, and **G** were calculated using Kruskal-Wallis test with Dunn’s multiple-comparison test or, in **B**–**E**, using Friedman’s test with Dunn’s multiple-comparison test and are shown above horizontal lines spanning comparison groups when significant. Lines bisecting groups represent median with IQR.

Since strong ADCC induced by hybrid immunity reflects the quality of immune response rather than simply abundance of IgG, we investigated the specificity of anti-S Abs following vaccination or hybrid immunity in more detail. Circulating IgG Abs against FLS and the individual S1 and S2 domains were measured by ELISA after infection as well as after first and second vaccinations. Anti–FLS IgG Abs were detected (optical density [OD] > 0.1) from 30 of 31 of the participants included in the hybrid cohort, and 38 of 40 of the vaccinated individuals (Figure 4A). Within the hybrid cohort, levels of anti-FLS Abs rose significantly after first vaccination, but there was no further increase upon second vaccination (Figure 4A). In contrast, Ab levels for the vaccinee cohort increased significantly following the second vaccination but remained relatively low (Figure 4A).

Infection generated IgG Abs against both S1 and S2 domains; however, anti-S2 Ab responses were favored over anti-S1 (Figure 4B). Vaccination after infection increased anti–S1 IgG Ab levels but had no significant impact on the levels of anti–S2 IgG Abs, indicating preferential boosting of anti-S1 Ab (Figure 4B). Similar skewing toward anti-S1 responses following vaccination was also apparent among those vaccinated with no prior infection, since substantially higher levels of anti–S1 IgG Abs compared with anti–S2 IgG Abs arose after both first and second vaccinations (Figure 4C). Thus, the anti–S IgG Ab response differs following infection or vaccination, with vaccination selectively inducing anti–S1 IgG Abs, while infection-induced immunity promoted higher levels of anti–S2 IgG Abs.

### Infection history dictates anti–S2 IgG_3_ Ab responses.

In addition to measuring the proportion of total IgG targeting S, we assessed the prevalence of IgG_3_ Abs in people with hybrid or vaccine-induced immunity. Anti–S1 IgG_3_ Ab levels after infection were generally low, with only 4 of 31 participants having OD > 0.1 (Figure 4D), but levels increased after first vaccination, with anti–S1 IgG_3_ Abs detected in 11 of 31 participants (Figure 4D). In contrast, anti–S2 IgG_3_ Ab levels were more substantial following infection, with 21 of 31 of participants having detectable anti–S2 IgG_3_ Abs (Figure 4D), and vaccination further improved anti–S2 IgG_3_ Ab levels (Figure 4D). With hybrid immunity, levels of anti–S2 IgG_3_ Abs were significantly higher than anti–S1 IgG_3_ Abs and remained stable for at least 5 months.

Vaccination alone resulted in 28 of 40 individuals producing anti–S1 IgG_3_ Abs after first vaccination and 31 of 40 after second vaccination (Figure 4E). One vaccination induced low amounts of anti-S2 IgG_3_ Abs in 16 of 40 persons, and levels improved after second vaccination, with 30 of 40 participants having detectable anti-S2 IgG_3_ (Figure 4E). Compared with hybrid immunity, levels of anti–S1 IgG_3_ Abs outweighed anti–S2 IgG_3_ following a single vaccination, and levels were similar following 2 vaccinations (Figure 4E). Therefore, for both total IgG and IgG_3_, infection-induced immunity favored anti-S2 Ab production, vaccination induced more robust anti-S1 Ab levels, and hybrid immunity imparted robust levels of both (Figure 4, F and G). The strongest IgG Ab responses against both S1 and S2 were seen following hybrid immunity.

### ADCC potency depends on Abs reactive against both S1 and S2.

**Figure 5:**
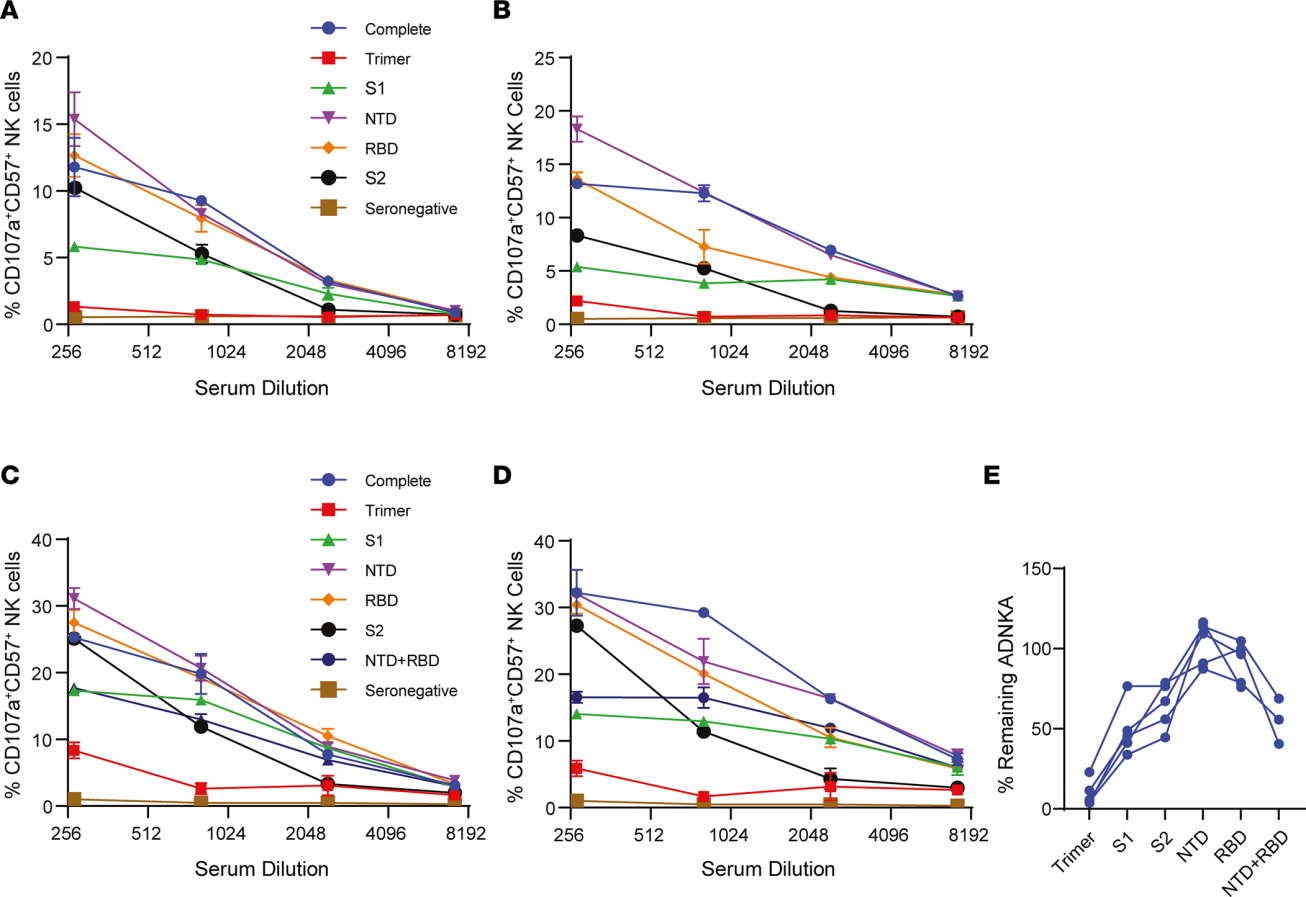
Assessment of ADNKA after S subunit Ab depletion. (**A**–**D**) Sera from individuals with hybrid immunity (PV2; *n* = 5) were diluted 1:9 and then Abs targeting the indicated domains of S were depleted using magnetic bead–conjugated protein. These depleted sera were then used to measure CD57^+^ NK cell CD107a expression at the indicated dilutions in the presence of SARS-CoV-2–infected A549-ACE2. Four individuals are depicted. (**E**) The AUC for each sample in **A**–**D** was calculated; then, the amount of ADNKA remaining following depletion of Abs targeting each domain in relation to sera containing all Abs, or sera from which all S Abs that had been depleted, was determined (*n* = 5). Lines bisecting groups in **A**–**D** represent mean ± SD.

To determine whether Abs targeting either or both of the S1 and S2 domains were responsible for potent S-specific ADCC following hybrid immunity, we selectively depleted Abs that target different S domains and assessed ADNKA against infected cells. ADNKA induced by vaccinee serum was too low to allow for observation of clear effects following depletion, so we focused solely on donors with hybrid immunity. Using 2 donors, we depleted Abs targeting the entire S trimer, the S1 domain, the S2 domain, or specific NTD or RBD domains found within S1. Sera were prediluted such that the starting dilution was sufficient to have diluted out Abs targeting antigens other than S (Figure 1C). Although depleting Abs targeting NTD or RBD alone led to only minor reductions in ADNKA, complete S1 or S2 subunit Ab depletion led to significant reductions in both donors (Figure 5, A and B), indicating that a combination of Abs targeting S1 and S2 support robust ADNKA.

Depleting Abs targeting NTD or RBD yielded smaller reductions in ADNKA than depleting Abs targeting the entire S1 domain. The reagents used for NTD and RBD depletion targeted amino acids 16–318 and 319–541, respectively, while the S1 depletion targeted amino acids 16–685. Since the S1 depletion potentially removed Abs that targeted amino acids 542–685, which were not depleted by RBD or NTD depletion, we repeated this experiment to include a double depletion of both NTD and RBD (Figure 5, C and D). The RBD/NTD double depletion closely mirrored S1 depletion. Combined data from 5 donors indicate that potent ADNKA induced by hybrid immunity was dependent on the presence of Abs reactive to both the S1 and S2 regions, as depletion of either region led to a significant loss in ADNKA (Figure 5E).

### Ab reactivity against 3 determinants along S correlates with potent ADCC.

**Figure 6:**
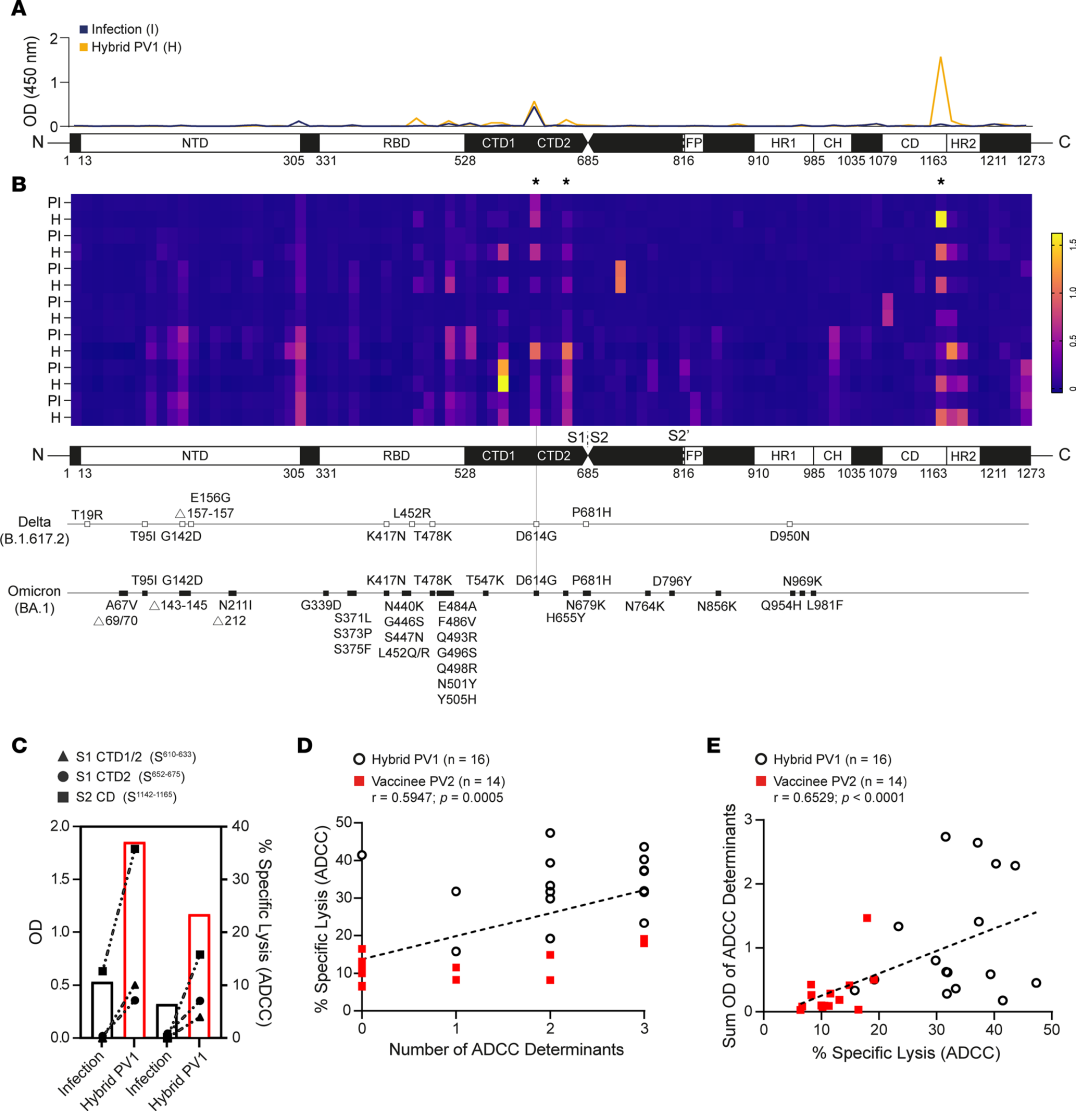
Peptide scanning to identify distinct linear regions associated with robust ADCC. Linear FLS Ab epitope reactivity was determined by ELISA-based peptide scanning. (**A**) A representative depiction of anti-IgG Ab reactive to linear segments is illustrated by an overlaid line graph. The blue line represents OD results of the full S peptide scan of a sample collected from 1 participant recovered from moderate COVID-19 infection, and the orange overlay represents a sample from the same participant collected 1 month after their first vaccination. (**B**) Compiled peptide scan data from samples collected from 7 participants postinfection (PI) and after hybrid immunity (H) were illustrated using a heatmap and aligned with known mutations in Delta and Omicron sequences. Three determinants associated with ADCC are identified by asterisks. (**C**) The left axis depicts anti–S1 CDT1/2 (triangle), –S1 CTD2 (circle), and anti–S2 CD (square) IgG Ab OD and compares these levels with ADCC (right axis) for 2 participants after infection (open black bar) and after subsequent vaccination (open red bar). (**D** and **E**) Participant IgG Ab OD collected from peptide scanning (*n* = 30) were scored for number of distinct regions or tallied, and associations between Ab reactivity and levels of ADCC were assessed by Spearman’s correlation.

Having determined that Abs against both S1 and S2 subunits were important for eliciting strong S-specific ADCC, we investigated the fine specificity of Ab responses arising from hybrid immunity by ELISA-based peptide scanning. In total, 181 overlapping peptides were coated onto ELISA plates in sequential pairs, and IgG Ab reactivity was measured. A comparison of Abs reactive to linear regions contained in both S1 and S2 produced after infection (Figure 6A, blue line) and subsequent vaccination (i.e., 2 antigen exposures; Figure 6A, orange line) revealed differential patterns and robustness of Ab reactivity. A heatmap illustrating changing patterns of linear Ab epitope reactivity along S before and after first vaccination is depicted for 7 participants in the hybrid group (Figure 6B).

Samples obtained after infection alone had Abs recognizing fewer S1 and S2 epitopes than samples collected from the same individuals following vaccination (Figure 6B), with Ab reactivity against 3 distinct regions along S particularly enriched among the hybrid samples (Figure 6B). These 3 areas of reactivity are contained within the (a) C-terminal domain (CTD) 1 and (b) CTD2 of S1 as well as (c) a region in S2 immediately upstream of the heptad repeat 2 (HR2) sequence in the connector domain (CD) (Figure 6B). Besides the early arising and common D614G mutation, these regions showed no genetic variation between Delta or Omicron variants (Figure 6B).

To illustrate potential associations between ADCC and Ab reactivity against these 3 regions, we used data collected from 2 people with infection-induced immunity and subsequent vaccination to depict the relationship between ADCC and the magnitude of anti-IgG Ab found in CTD1/2 and CD. In both cases, increasing anti-IgG Ab levels specific for each of the 3 regions paralleled robust increases in ADCC (Figure 6C). Furthermore, there were significant associations between ADCC and both (a) the number of ADCC determinants to which a participant demonstrated IgG Ab reactivity (Figure 6D) and (b) the cumulative OD from the 3 distinct regions (Figure 6E). Thus, epitope specificity plays a key role in S-specific killing of SARS-CoV-2–infected cells.

### Hybrid immunity broadly mediates ADCC across variant strains.

**Figure 7:**
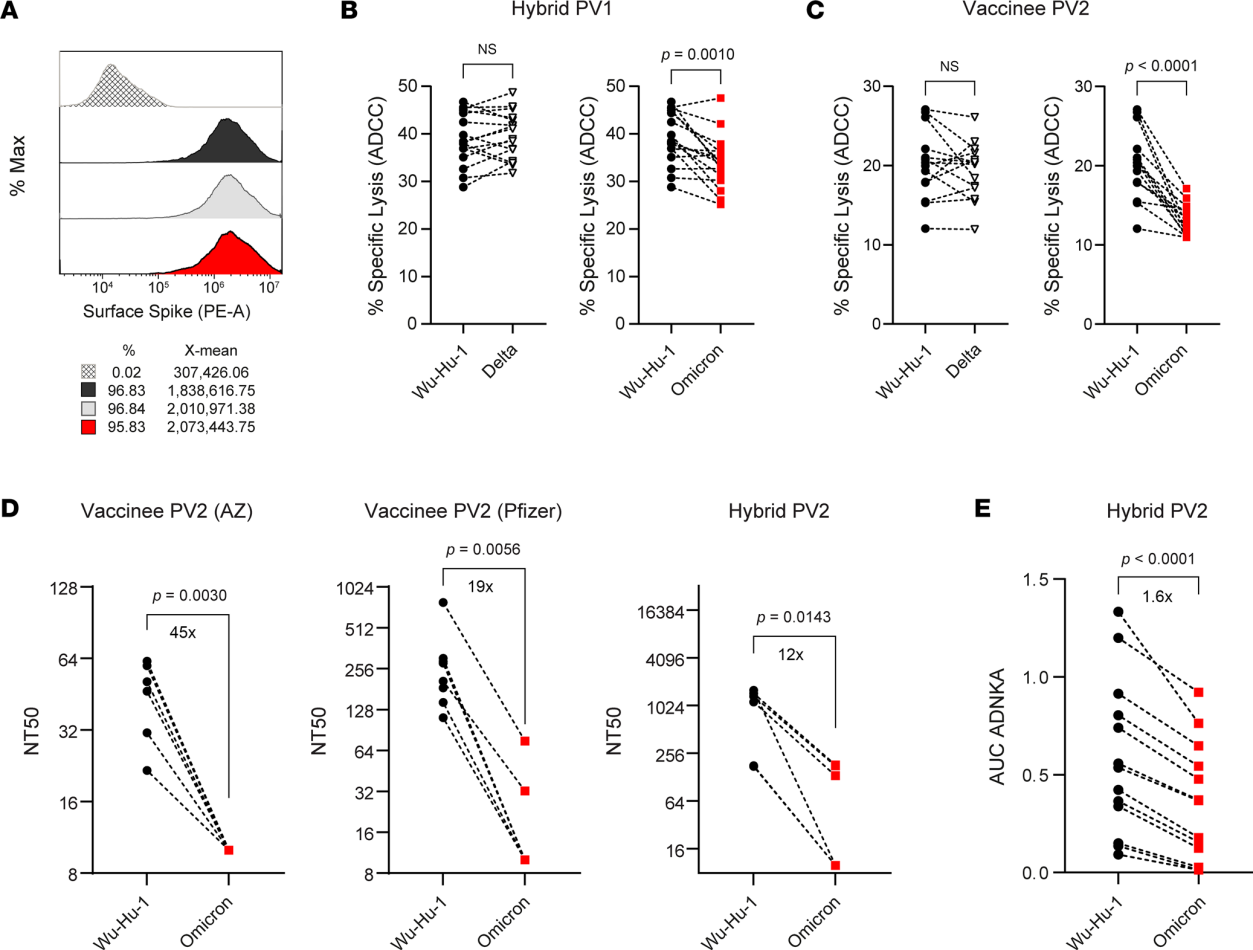
Vaccine- and hybrid-induced S-specific ADCC against variants of concern. (**A**–**C**) Histogram overlay of MRC-5 cells transduced to express similar levels of Wuhan-Hu-1 (black), Delta (gray), and Omicron (red) S protein used in ^51^Cr assay to assess the efficacy of Ab produced with hybrid (*n* = 14) or vaccine-induced (*n* = 16) immunity in eliciting ADCC against variant strains. Experiments were performed in duplicate with 3 independent donors, and a representative plot is shown. (**D**) SARS-CoV-2 neutralization assay was performed on serial dilutions of serum samples from vaccinees (*n* = 13) or persons with hybrid immunity (*n* = 5), and NT50 values against either Wuhan-Hu-1 or Omicron-infected A549-ACE2 were calculated. (**E**) Serum samples were serially diluted, and CD57^+^ NK cell CD107a expression against A549-ACE2 cells infected with either Wuhan-Hu-1 or Omicron was measured by flow cytometry; then, AUC was calculated. *P* values in **B**–**E** were calculated using Student’s paired *t* test and shown above horizontal lines spanning comparison groups when significant.

Virus evolution in human populations results in selection of viral variants mutated at key residues for neutralizing Ab binding — primarily within the RBD but also within the NTD (7, 8). However, ADCC-susceptible determinants are distributed much more broadly across S (Figure 5), and ADCC determinants revealed by peptide scanning do not overlap with mutations in recent virus variants such as Delta and Omicron (Figure 6B). This suggests that ADCC induced by hybrid immunity may be better preserved than neutralization across variant strains. To assess S-directed FcR–dependent responses against variant strains, MRC-5 cells were transduced to express Delta or Omicron S at levels equivalent to Wu-S–MRC-5 (Figure 7A). When used in ADCC assays, Abs produced after 2 antigen exposures with hybrid immunity (i.e., infection and 1 vaccination) elicited comparable levels of ADCC against target cells expressing Wuhan-Hu-1 and Delta S (Figure 7B). Although ADCC was reduced against Omicron S–expressing cells, the relative reduction was mild (12.9% decrease), and 5 of 16 participants mediated equivalent levels of ADCC against cells expressing Wuhan-Hu-1, Delta, and Omicron S (Figure 7B).

A subset of vaccinees mediating detectable ADCC against Wu-S–MRC-5 were similarly tested. Here, despite also having 2 antigen exposures, the decline in ADCC against cells expressing Omicron S was more pronounced with a 35.5% relative reduction (Figure 7C). Abs from only 1 of 14 vaccinees mediated comparable levels of ADCC against WT and variant strains (Figure 7C), consistent with Abs in these donors being focused on the S1 domain, which is more heavily mutated in these virus variants.

To ensure that these results were comparable with an infection setting, we assessed neutralization and ADCC against replicating virus using either ancestral Wuhan-Hu-1 or Omicron strains. Consistent with published data, we observed that Omicron was more resistant to neutralization following vaccination, and despite this decrease in neutralization being less severe following hybrid immunity compared with vaccination, a significant loss was observed (Figure 7D). In contrast, and in agreement with data obtained using cells overexpressing S, we measured only a small decline in ADNKA against Omicron-infected cells (Figure 7E). Thus, by targeting a broader range of epitopes, including those more conserved across variants, ADCC resists virus escape due to virus mutation more effectively than neutralization.

## Discussion

Strong and durable immune responses are desirable to limit SARS-CoV-2 transmission and control severity of infection. Neutralizing Ab activity has dominated as a surrogate measure of protection; however, protection against severe illness without robust neutralization suggests that other aspects of immunity, including T cells and NK cells, play a role (38). The ability of T cells and NK cells to limit illness by eliminating infected host cells once infection does occur underlies the importance of considering their recruitment in vaccination strategies, especially if their activity is better conserved across variants than Ab neutralization (39, 40).

Associations between ADCC and viral control in animal models (12–22) indicate that ADCC is an important component of immunological protection. By targeting infected cells directly, ADCC should effectively limit virus spread regardless of whether dissemination occurs through cell-to-cell contact or extracellular release. Infection with SARS-CoV-2 induces Abs against N/M/ORF3a that dominate NK cell activation by infected cells (11); however, in the absence of vaccines encoding alternative ADCC antigens, it is important to understand the capacity for S-specific ADCC. Infection or vaccination alone was insufficient to induce potent S-directed ADCC, whether assessed by direct killing of S-expressing cells or by NK cell activation against SARS-CoV-2–infected cells. However, the strong ADCC observed with hybrid immunity indicates that it is possible to generate robust and lasting ADCC through S-targeted Ab responses.

Previous analysis of hybrid immunity in the context of neutralizing Abs demonstrated that, compared with vaccination alone, hybrid immunity results in more memory B cells and circulating Abs, the latter of which was affirmed in our study (41, 42). This increase in circulating IgG enhances neutralization of more immune-evasive virus variants (41, 43, 44), while at the clonal level, it also reflects selection of Abs with higher neutralizing potency (45). In the case of ADCC, neither increased abundance nor differences in IgG subclass could explain superior hybrid immunity–induced ADCC. Instead, qualitative aspects of the Ab profile played a more important role. Furthermore, robust responses were not dependent on the number of antigen exposures but rather on the nature of antigen exposure. Abs eliciting strong and durable ADCC were generated after only 2 antigen exposures in people with hybrid immunity (1 vaccination), and there was no further advantage after 3 exposures (2 vaccinations), clearly contrasting with the weak ADCC elicited by Abs from vaccinees after the same number of antigen exposures through vaccination alone (both 2 and 3 doses).

Multiple parameters influence NK cell activation upon FcR crosslinking, including Ab density, isotype, affinity, and specificity (36). Greater levels of Abs targeting epitopes in both S1 and S2 were consistent features of hybrid immunity and robust ADCC, indicating that Ab specificity plays a critical role. Abs bound to membrane-proximal epitopes induce potent ADCC, presumably by decreasing the size of the immune synapse or better promoting FcR clustering (46). Thus, since they bind closer to the target cell membrane, Abs against the S2 stalk domain may have a substantial role in the ADCC advantage noted with hybrid immunity. Robust ADCC may also benefit from coordinated binding of anti-S1 and anti-S2 Abs to facilitate FcR clustering. Studies in the context of HIV and influenza A virus (IAV) have demonstrated that Abs recognizing distinct but proximal cognate epitopes positively modulate FcR crosslinking and the magnitude of ADCC (47, 48). Although synergistic FcR engagement by different Abs recognizing the same glycoprotein was required for effective HIV-specific ADCC, competition between IAV hemagglutinin stalk–binding Abs and Abs binding the head domain actually impaired IAV-specific ADCC (47, 49). Identifying and isolating anti-S Abs capable of synergizing to induce robust ADCC against SARS-CoV-2 will be needed to help researchers understand whether similar phenomena underpin our observations. Our previous screen of anti-S mAbs did not identify any that were capable of inducing robust ADCC alone or when up to as many as 5 were combined (11). This suggests that either anti-S mAbs inducing robust ADCC are rare or that ideal polyclonal combinations are required to effectively crosslink FcR to mediate robust S-specific ADCC.

The S1 domain of S contains the NTD and RBD domains, which are required for receptor binding and are dominant targets for neutralizing Abs. As a result, vaccine development — including the testing of RBD subunit vaccines — has focused heavily on inducing S1-targeted Abs (50–54). However, due to mutations selected in S1 that tend to reduce neutralization, there is increasing interest in neutralizing Abs targeting the more conserved S2 domain (55). Studies in mice demonstrated that S2-specific vaccination effectively induces cross-variant neutralization (55). Our work now extends this by showing that strategies to induce Abs against epitopes within both S1 and S2 will also serve to broadly boost ADCC against multiple SARS-CoV-2 variants. The polyspecific (i.e., S1 and S2) nature of S-directed ADCC improves targeting of virus variants with heavily mutated S1 and may limit the ability of viruses to evade this arm of host defense. With a wider range of Abs recognizing epitopes distributed throughout S, the loss of any single epitope may not substantially impact ADCC, thereby raising the barrier for escape mutant selection.

The S gene of seasonal human β coronaviruses (HCoV HKU1, HCoV OC43) is divergent from SARS-CoV-2 S, sharing only ~ 36% overall homology, with the S2 domain somewhat more conserved than S1. The preferential Ab targeting of S2 epitopes after infection may be partly attributed to a heterologous boost toward cross-reactive S2-specific memory B cells (56–58), with preexisting cross-reactive HCoV memory B cells activated during SARS-CoV-2 infection kick-starting production of anti-S2 Abs. Interestingly, the same regions we revealed as key regions of Ab reactivity corresponding with potent ADCC were previously demonstrated to be elicited by heterologous anti-S Ab responses (58). With 75% sequence homology between the major SARS-CoV-2 determinant in S2 and HCoV OC43, this raises the question as to whether immunodominant regions within S1 and S2 conserved between HCoV and SARS-CoV-2 are selectively targeted for Ab reactivity and avidity to support robust ADCC. If SARS-CoV-2 infection induces cross-reactive HCoV anti-S2 Ab production, it is unclear why vaccination did not have a similar effect. Prolonged antigen exposure from high viral loads, the presence of inflammatory stimuli during virus infection, the way S traffics to and is presented on the infected cell surface, or differential sites of antigen presentation (e.g., intramuscular for the vaccine, respiratory for the virus) may all contribute to diverse outcomes from exposure through infection versus S subunit vaccination. It will be interesting to see whether switching vaccine delivery from intramuscular to intranasal administration, to promote mucosal immunity, impacts this process (59, 60).

This study focused on individuals who were infected prior to vaccination. Many individuals have now been infected after being vaccinated with ancestral S antigen, and it is unclear how these breakthrough infections will affect ADCC against emerging variants. Our previous research demonstrated that infection induces non-S ADCC responses that target N/M/ORF3a (11). However, since vaccination reduces the induction of non-S (e.g., N) Abs during breakthrough infection, presumably due to enhanced vaccine-mediated immunological control (61–63), breakthrough infections may fail to induce potent N/M/ORF3a-mediated ADCC. In this case, strong S-directed ADCC induced by hybrid immunity would become an important effector mechanism contributing to protection and an important consideration for future research.

In summary, our data reveal that hybrid immunity establishes conditions whereby Abs generated against key determinants within S1 and S2 domains elicit ADCC quantitatively superior to vaccination or infection alone. In addition, hybrid immunity induced by ancestral antigen engenders an Ab response that retains activity against variant strains to a far greater extent than neutralization. Given the complementary roles of neutralization and ADCC in controlling cell-free and cell-associated virus, respectively, both Fc-region– and Fab V-region–mediated effector functions are desirable for incorporation into vaccine strategies. Strong ADCC may play a part in the protection offered by hybrid immunity, may uncover a novel role for S2-targeted Abs, and suggests that vaccine strategies based on S expression would benefit from inducing Abs targeted broadly across S, as opposed to just RBD.

## Methods

### Participants.

This study was carried out at 2 sites. In Canada, 31 individuals with confirmed infection who continued in the study and received 2 doses of a COVID-19 vaccine were matched with 40 individuals with no previous infection history who had received 2 doses of a COVID-19 vaccine (Table 1). Asymptomatic participants were identified through public health surveillance and contact tracing after contact with reverse transcription PCR–confirmed (RT-PCR–confirmed) cases or through serological testing for anti–S and anti–N protein IgG Abs (64). Most participants enrolled following the first wave of COVID-19, and infections were attributed to ancestral SARS-CoV-2 isolate Wuhan-Hu-1. Three participants had infections attributed to B.1.1.7. Participants self-declared any medical treatments they were receiving as well as information on comorbidity. Persons with any known underlying immune-compromising condition or on immunosuppressive treatment were excluded.

In the United Kingdom, peripheral blood was collected 2–4 weeks after a RT-PCR or LFD confirmed infection, or after vaccination, from otherwise healthy donors. Alternatively, serum samples submitted as part of the Avon Longitudinal Study of Parents and Children (ALSPAC) cohort (65–68) were assessed based on serology for N and S in order to identify individuals who had submitted samples after an initial infection, followed by those who had submitted samples after each vaccination. In total, 18 individuals who were infected prior to vaccination were matched with 18 individuals who had received 2 doses of vaccines with no experience of prior infection. Results were also compared with samples from a previously described cohort of individuals who had experienced mild or severe COVID-19 and had not been vaccinated (11).

### Blood sample processing.

In Canada, whole blood was collected by venipuncture in acid citrate dextrose vacutainers, after which plasma was collected following 10 minutes of centrifugation at 500*g* at room temperature and stored at –80°C. Alternatively, in the United Kingdom, whole blood was collected in a serum-separating vacutainer and serum collected following centrifugation at 500*g* for 10 minutes at room temperature. PBMC used for ADCC experiments were isolated from anticoagulated heparinized blood from healthy donors by density gradient centrifugation using the Canadian Autoimmunity Standardization Core consensus standard operating procedure (version: March 21, 2019; https://www.bcchr.ca/CAN-ASC/protocols). Freshly isolated PBMC were resuspended in lymphocyte medium consisting of RPMI-1640, 10% FBS (HyClone), 200 IU/mL penicillin/streptomycin, 0.01M HEPES, 1% L-glutamine (all from Invitrogen), and 2 ***×*** 10^–5^ M 2-mercaptoethanol (Sigma-Aldrich) and then used directly in functional experiments.

### Cell lines and viruses.

All cell lines and PBMC were cultured with 5% CO_2_ at 37°C. VeroE6 cells expressing ACE2 and TMPRSS2 (VAT) and A549 cells expressing ACE2 (AA) were a gift from the University of Glasgow Centre for Virus Research (Glasgow, United Kingdom) (69). Human lung fibroblast MRC-5 cells were obtained from ATCC (CCL-171), and Lenti-X 293T cells were obtained from Takara. All were propagated in complete DMEM (Sigma-Aldrich) containing 10% FBS (HyClone) and 200 IU/mL penicillin/streptomycin (Invitrogen). Ancestral SARS-CoV-2 was recovered from a BAC containing the complete genome of a strain that matches the original Wuhan-Hu-1 (69), and the Omicron (BA.1) variant was a gift from Arvind Patel (University of Glasgow Centre for Virus Research). Both were propagated in VAT cells. Virions were concentrated and purified by pelleting through a 30% sucrose cushion and titrated by plaque assay in AA cells, as previously described (11). All virus seed stocks were verified by whole-genome sequencing on the Illumina platform.

### S gene transfer and expression in MRC-5 cells.

The recombinant Lenti-X pLVX-IRES lentiviral vector expression system (Takara) was used to introduce Wuhan-Hu-1, Delta (B.1.617.2), or Omicron (BA.1) S sequences into the MRC-5 cell line. The Wuhan-Hu-1 S was obtained from BEI Resources in a pcDNA3.1(-) mammalian expression vector (NR-52420; NIAID, NIH) (70). Delta S was synthesized by Invitrogen GeneArt (Thermo Fisher Scientific) and contains the following mutations: T19R, T95I, G142D, E156G, E157- F158- R450L, K476T, G612D, R679P, and N948D. Omicron S was obtained from BEI Resources in a pCMV/R mammalian expression vector (NR-56470; NIAID, NIH) with the following mutations: A67V, H69del, V70del, T95I, G142del, V143del, Y144del, Y145D, N211del, L212I, “EPE” insertion between 214R and 215D, G339D, S371L, S373P, S375F, K417N, N440K, G446S, S477N, T478K, E484A, Q493R, G496S, Q498R, N501Y, Y505H, T547K, D614G, H655Y, N679K, P681H, N764K, D796Y, N856K, Q954H, N969K, and L981F.

Constructs were inserted into pLVX-IRES as detailed previously (71) using conventional cloning protocols, and all constructs were verified by forward and reverse strand sequencing (TCAG Facilities, Hospital for Sick Children, Toronto, Ontario, Canada) to ensure authenticity. SnapGene Software was used for designing and visualizing cloning procedures, designing and aligning sequencing primers, and comparing variant sequences to Wuhan-Hu-1. Transduced cells were propagated and selected in complete DMEM containing 1 μg/mL puromycin (Sigma-Aldrich). Extracellular S expression was confirmed by flow cytometry, as previously outlined (71).

### Ab-dependent cell-mediated killing assays.

PBMC were freshly processed, resuspended in lymphocyte medium, and kept at 37°C and 5% CO_2_ until use. For ADCC assays using Wuhan-Hu-1–S–, Delta S–, or Omicron S–expressing MRC-5 target cells, 1 ***×*** 10^4^ cells/well were plated and labeled with 1 μCi Na_2_^51^CrO_4_/well (PerkinElmer) overnight in 96-well round-bottom plates and then washed 4 times in PBS containing 1% FBS (HyClone). PBMC (E:T, 25:1) and heat-inactivated plasma (56°C for 1 hour) were added to wells with a final volume of 300 μL and final plasma dilution of 1:1,000. Cytotoxic activity was measured by ^51^Cr release over 5 hours. ^51^Cr release was measured in 125 μL of supernatant on a Wallac 1480 Wizard gamma counter, and percent specific lysis was calculated by the following: (experimental ^51^Cr release – spontaneous ^51^Cr release)/(maximum ^51^Cr release – spontaneous ^51^Cr release) ***×*** 100.

Assays using virus-infected cells used CD107a degranulation ADNKA as a proxy for ADCC to be in compliance with BLS3 containment and were carried out as previously described (11). Briefly, AA cells were infected at MOI = 5 for 24 hours prior to being detached with TrypLE (Thermo Fisher Scientific)after which time 2.5 ***×*** 10^4^ targets were mixed with 2.5 ***×*** 10^5^ PBMC, serum, and anti–CD107a-FITC (H4A3, BioLegend) and GolgiStop (BD Biosciences) in a total volume of 100 μL for 5 hours. PBMC were stained with live/dead fixable aqua (Thermo Fisher Scientific), anti–CD3-PE-Cy7 (UCHT1, BioLegend), anti–CD56-BV605 (5.1H11, BioLegend), and anti–CD57-APC (HNK-1, BioLegend). Data were acquired and analyzed using an Attune NXT Flow Cytometer (Thermo Fisher Scientific) and expressed as the percentage of live CD107a^+^CD57^+^ NK (CD3^–^CD56^+^) cells. All sera were tested against mock-infected cells to ensure there was no background NK cell activation, and any sera demonstrating background activation were excluded. A seronegative serum was included in all assays as a negative control. To enable comparisons with previous data sets, and to minimize interexperiment variability, a donor serum demonstrating moderate ADNKA was included as a positive control in every assay. Sera were tested at a range of dilutions, and the AUC was calculated using GraphPad Prism 9. This value was then normalized to the AUC for the standard serum in each assay.

### Virus neutralization assay.

Assays were carried out as previously described (11). Briefly, 600 plaque-forming units of SARS-CoV-2 were incubated with appropriate dilutions of serum, in duplicate, for 1 hour at 37°C. The mixes were then added to preplated VeroE6 cells for 48 hours. After this time, monolayers were fixed with 4% paraformaldehyde (Thermo Fisher Scientific), permeabilized for 15 minutes with 0.5% NP-40 (Merck Life Science), and blocked for 1 hour in PBS containing 0.1% Tween (PBST) and 3% nonfat milk. Primary Ab (anti–N 1C7; clone 1C7; BSM-41411M; Stratech, 1:500 dilution) was added in PBST containing 1% nonfat milk and incubated for 1 hour at room temperature. After washing in PBST, secondary Ab (anti–mouse IgG-HRP; 715-035-151; Jackson ImmunoResearch, 1:3,000 dilution) was added in PBST containing 1% nonfat milk and incubated for 1 hour. Monolayers were washed again, developed using Sigmafast OPD (Sigma-Aldrich) according to manufacturers’ instructions, and read on a Clariostar Omega plate reader (OD, 450 nm). Wells containing no virus, virus but no Ab, and a standardized serum displaying moderate activity were included as controls in every experiment. The 50% neutralization titer (NT50) were calculated in GraphPad Prism 9.

### Serological testing.

Plasma was diluted in PBS containing 0.05% TWEEN 20 (0.05% PBST; Sigma-Aldrich) and 0.1% BSA (Sigma-Aldrich); it was then tested against recombinant proteins coated in Dulbecco’s PBS (DPBS, Sigma-Aldrich) overnight onto 96-well Immunlon-2 plates (VWR Scientific). Recombinant protein antigens included SARS-CoV-2 FLS glycoprotein trimer (50 ng/well; SMT1-1 reference material, National Research Council [NRC], Canada), the S1 subunit of SARS-CoV-2 S (65 ng/well; SinoBiological), and S2 subunit of SARS-CoV-2 S (50 ng/well; SinoBiological). The predicted molecular masses for S1 and S2 were 76.5 kDa and 59.4 kDa, respectively, and coating amounts were determined to account for this difference. Plates were washed 4 times with 0.05% PBST and then blocked for 1 hour with 200 μL of PBS containing 1% BSA (Sigma-Aldrich). After 4 washes, 100 μL/well of diluted plasma (1:500 for FLS IgG or 1:100 for S1 and S2 IgG and IgG_3_) was applied to antigen-coated plates in duplicate wells for 1.5 hours. Total IgG was measured following 6 washes and a 1-hour incubation with 100 μL/well of 1:50,000 HRP-conjugated polyclonal goat anti–human IgG (109-035-088; Jackson ImmunoResearch, 1:50,000 dilution). IgG_3_ was measured following 6 washes and a 1-hour incubation with 100 μL/well of 1:5,000 mouse anti–human biotin–conjugated IgG_3_ hinge (9210-08; SouthernBiotech, 1:5,000 dilution), followed by 6 washes and 1-hour incubation with 100 μL/well of 1:40,000 HRP-conjugated streptavidin (016-030-084; Jackson ImmunoResearch). Plates were developed using tetramethylbenzidine (TMB) substrate (Sigma Aldrich) following 6 washes; they were then incubated in the dark at room temperature for 20 minutes. Reactions were stopped with an equal volume of 1 M H_2_SO_4_, and OD was read on a BioTek synergy HT plate reader at 450 nm.

### Ab depletions.

Depletions of specific Abs from sera were carried out as previously described (11). In brief, Abs targeting different domains of S were depleted using magnetic bead–conjugated proteins based on either the RBD, NTD, entire S1, or entire S2 domain of SARS-CoV-2 Wuhan-Hu-1 S (ACROBiosystems). Beads were resuspended in PBS + 0.05% BSA; then, serum was diluted 1:9, and 50 μL mixed with 150 μL beads. Mixtures were incubated on a rotating mixer at 4°C overnight. Serum diluted in buffer alone was used as a control. Magnetic beads were removed using a 3D-printed magnetic stand, followed by a second round of depletion using fresh beads. All values in assays were corrected for dilutions.

### Peptide scan ELISA.

Individual overlapping peptides (17 or 13 mers, with 10 aa overlaps) spanning the canonical Wuhan-Hu-1 S sequence (NR-52402; BEI Resources) were reconstituted at 10 mg/mL in DMSO (Sigma-Aldrich) and were then diluted to 50 μg/mL in DPBS (Sigma-Aldrich) and stored at –20°C. In total, 125 ng/well of each S peptide 2 – peptide 181 (BEI Resources) was coated overnight on Immunlon-2 plates (VWR Scientific) in sequential pairs (e.g., 2 and 3 … 180 and 181). The leader sequence (peptide 1) was coated onto a distinct well. FLS trimer (SMT1-1, NRC) was diluted in DPBS and coated overnight at 150 ng/well as positive control. Plates were washed 4 times with 0.05% PBST and blocked for 1 hour with 200 μL/well PBS + 1% BSA. Plasma was diluted 1:50 in 0.05% PBST + 0.1% BSA, and 50 μL was applied for 1.5 hours. Plates were washed 6 times, and total IgG binding was measured in a 1-hour incubation with 100 μL/well of 1:50,000 HRP-conjugated polyclonal goat anti–human IgG (109-035-088; Jackson ImmunoResearch, 1:50,000 dilution) and developed using 50 μL/well TMB substrate (Sigma Aldrich). Reactions were stopped with an equal volume of 1M H_2_SO_4_, and OD was read on a BioTek synergy HT plate reader at 450 nm.

### Statistics.

Statistical analyses were performed using GraphPad Prism 9 with 2-sided *P* < 0.05 considered significant. Normality of data distributions were assessed using Shapiro-Wilk test. Significance in correlations were assessed using Spearman’s rank correlation coefficient. Differences in means with SD or medians with IQR (calculated as IQR = Q_3_ − Q_1_) between groups were compared by using 1-way ANOVA, 2-tailed Student’s *t* test, Friedman test ,or Mann-Whitney *U* test as appropriate based on normality of data distribution.

### Study approval.

This study was carried out at 2 sites. In Canada, the study conformed to recommendations of the Canadian Tri-Council Policy Statement: Ethical Conduct for Research Involving Humans, and ethical approval was given by the Health Research Ethics Authority of Newfoundland and Labrador (HREB). Peripheral blood was collected from study subjects at approximately 3-month intervals, and a questionnaire addressing previous testing history and reasons for suspecting infection with SARS-CoV-2 was administered at study intake after written informed consent in accordance with the Declaration of Helsinki. In the United Kingdom, ethical approval was given by Cardiff University School of Medicine Research Ethics Committee or the ALSPAC Ethics and Law Committee, and consent for biological samples was collected in accordance with the Human Tissue Act (2004). Additional information for ALSPAC ethical approvals: http://www.bristol.ac.uk/alspac/researchers/research-ethics/ The ALSPAC study website contains details of all data available through a fully searchable data dictionary and variable search tool: http://www.bristol.ac.uk/alspac/researchers/our-data/

### Data availability.

Data supporting the findings of this study are available in the Supporting Data Values (supplemental material available online with this article; https://doi.org/10.1172/jci.insight.170681DS1). 

## Author contributions

KAH, RJS, CAF, and KB designed and conducted the experiments, analyzed data, constructed figures and illustrations, and wrote/edited the manuscript. MDG and ECYW cowrote and edited the manuscript. KMH and DPI assisted with experiments. DH coordinated patient consent and questionnaires and collected blood samples. MDG, ECYW, RJS, and KAH obtained funding.

## Supplementary Material

Supporting data values

## Figures and Tables

**Table 1:**
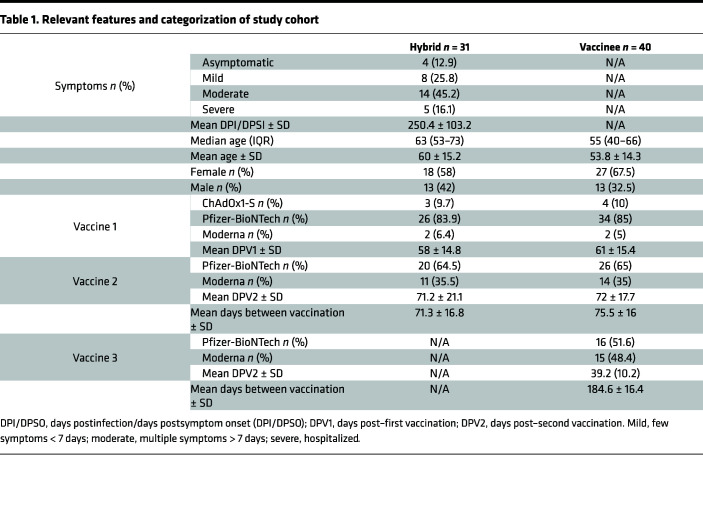
Relevant features and categorization of study cohort

## Version 1 06/20/2023

In-Press Preview

## Version 2 08/08/2023

Electronic publication
